# Supplementary material for: Initial Virome Characterization of the Common Cnidarian Lab Model Nematostella vectensis
Source: Viruses. 2020 Feb 15;12(2):218. doi: 10.3390/v12020218 (PMC7077227; doi:10.3390/v12020218)

▼File: Untitled1.syz

IMPORT successfully completed.

▼Factor Analysis

Latent Roots (Eigenvalues)

1	2	3	4	5	6
3.161	2.278	0.389	0.151	0.014	0.006

Component Loadings

	1	2	3	4	5
**C2**	0.754	-0.635	0.005	0.156	-0.029
**C3**	0.820	-0.444	0.199	-0.301	0.000
**C4**	0.592	-0.774	-0.189	0.110	0.029
**C5**	0.659	0.561	-0.491	-0.097	0.009
**C6**	0.764	0.625	0.115	0.071	-0.078
**C7**	0.743	0.610	0.243	0.099	0.079

Variance Explained by Components

1	2	3	4	5
3.161	2.278	0.389	0.151	0.014

Percent of Total Variance Explained

1	2	3	4	5
52.690	37.968	6.484	2.522	0.235

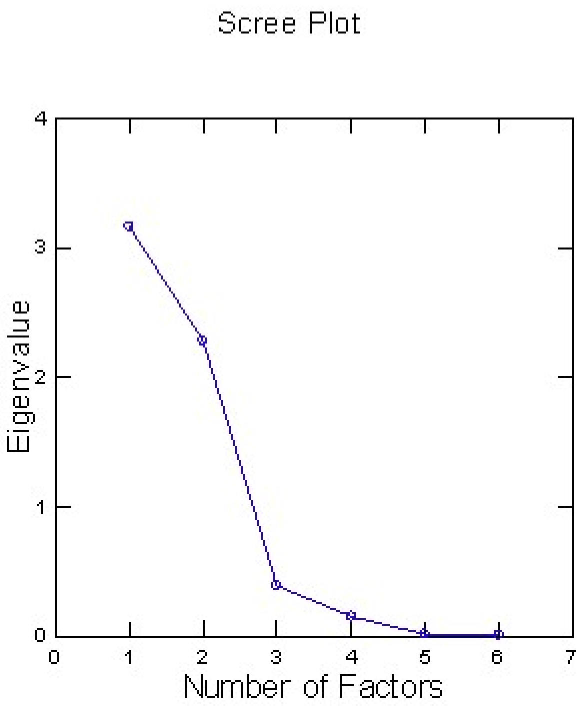

Factor Loadings Plot

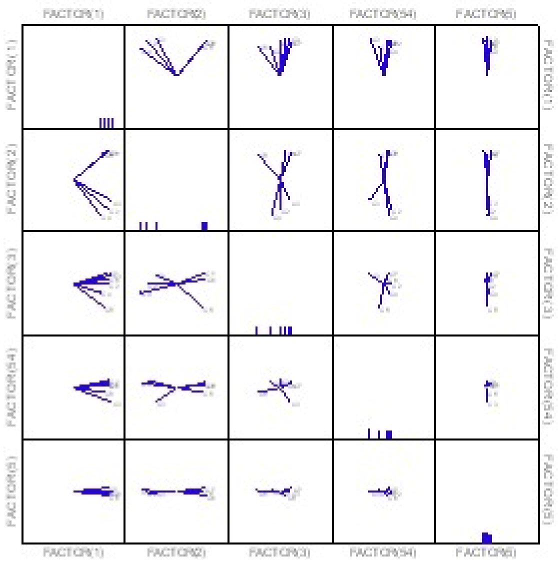

►Factor Analysis
▼Hypothesis Testing: Two-sample t-test

Two-sample t-test on PCA1 Grouped by PROJECT vs Alternative = 'not equal'

GROUP	N	Mean	Standard Deviation
1	3	0.722	0.117
7	3	0.722	0.056

Separate Variance

Difference in Means	:	0.000
95.00% Confidence Interval	:	-0.246 to 0.246
t	:	0.000
df	:	2.854
p-value	:	1.000

Pooled Variance

Difference in Means	:	0.000
95.00% Confidence Interval	:	-0.208 to 0.208
t	:	0.000
df	:	4.000
p-value	:	1.000

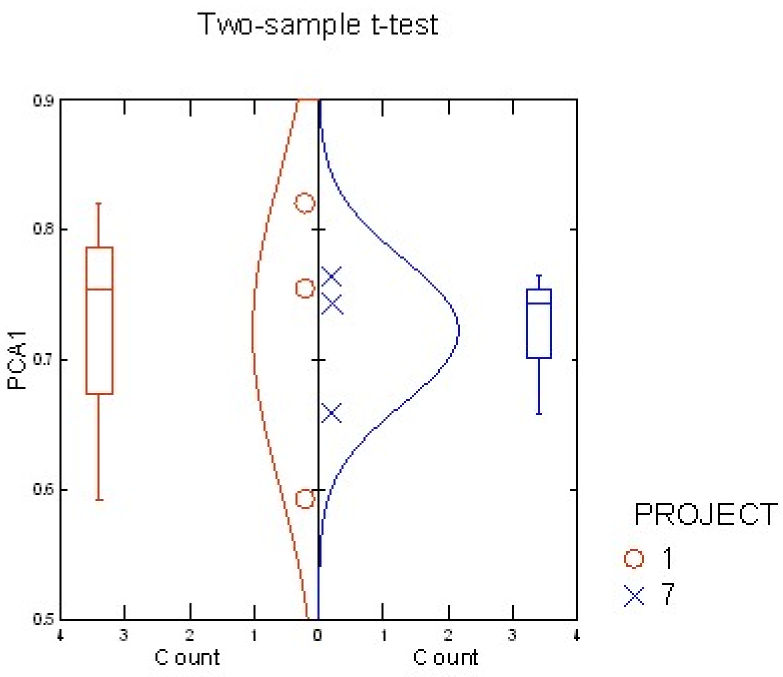

▼Hypothesis Testing: Two-sample t-test

Two-sample t-test on PCA2 Grouped by PROJECT vs Alternative = 'not equal'

GROUP	N	Mean	Standard Deviation
**1**	3	-0.616	0.168
**7**	3	0.599	0.033

Separate Variance

Difference in Means	:	-1.215
95.00% Confidence Interval	:	-1.611 to -0.819
t	:	-12.300
df	:	2.159
p-value	:	0.005

Pooled Variance

Difference in Means	:	-1.215
95.00% Confidence Interval	:	-1.489 to -0.941
t	:	-12.300
df	:	4.000
p-value	:	0.000

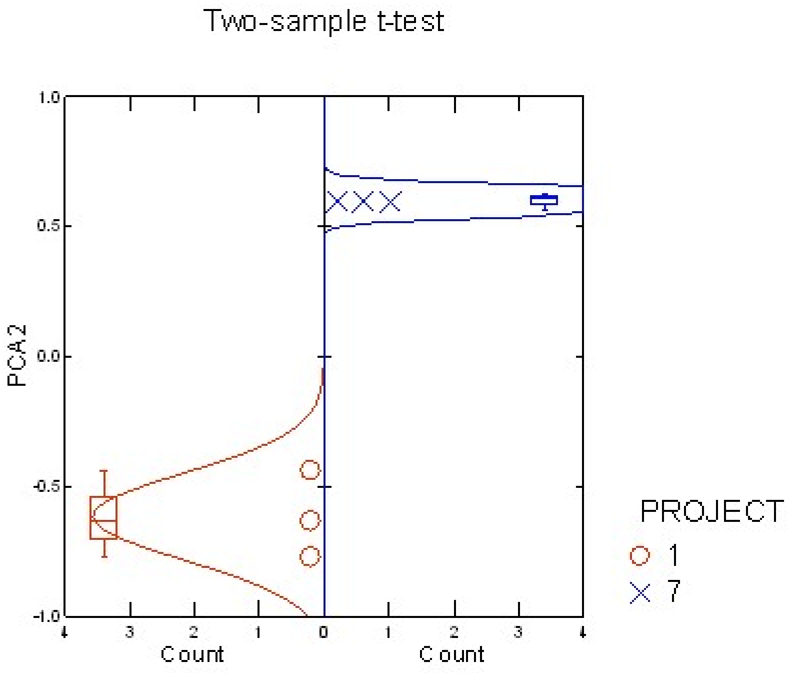

Supplement: Supplementary file 1 [file viruses-12-00218-s001.zip › Supplementary file 2.pdf]
